# The effects of different root canal irrigation protocols and artificial aging procedures on the bond strength between dentin and hybrid ceramic posts

**DOI:** 10.1186/s12903-022-02571-x

**Published:** 2022-12-09

**Authors:** Celalettin Topbaş, Şevki Çınar, Bike Altan, Dursun Ali Şirin, Mehmet Ali Fildişi

**Affiliations:** 1 Department of Endodontics, Faculty of Dentistry, University of Health Sciences, +905055993249 Uskudar, Istanbul, Turkey; 2 Department of Prosthodontics, Faculty of Dentistry, University of Health Sciences, Istanbul, Turkey; 3 Department of Endodontics, Faculty of Dentistry, University of Health Sciences, Istanbul, Turkey; 4 Department of Restorative Dentistry, Faculty of Dentistry, Medipol University, Istanbul, Turkey

**Keywords:** EDTA, Maleic acid, Micro-pushout bond strength, Vita Enamic, Post

## Abstract

The purpose of this study is to investigate the effects of different root canal irrigation protocols applied to the dentin and artificial aging procedures on the micro pushout bond strength (mPBS) between dentin and hybrid ceramic posts. Seventy-five single-rooted mandibular premolar teeth were divided into 5 groups (Gr1-5). 50 of the teeth were used for the mPBS tests (*n* = 10), whereas 25 were used for the smear layer examinations (*n* = 5). Post space were prepared and irrigated with different irrigation-protocols in each group. (Gr1:[SS], Gr2:[NaOCl] + SS, Gr3:[EDTA] + NaOCl + SS, Gr4:[MA] + NaOCl + SS, Gr5:[Ch] + NaOCl + SS). Post and core pattern were fabricated with pattern resin and a fiber post, after scanning, the posts were milled with Vita Enamic resin ceramic block, and cemented. After 7 days the roots were sliced at thicknesses of 1 mm; half of them were subjected to mPBS test, while the other half were tested after undergoing mechanical cycling for artificial aging. For data analysis, the Shapiro-Wilk test was utilized to test normal distributions, 3-way analysis of variance was used to compare mPBS, and Tukey’s HSD test was conducted for multiple comparisons. SEM analysis was performed for examination of failure modes and smear layer removal. Different root canal irrigation protocols affected mPBS significantly. While Gr4 had the highest mPBS, Gr1 had the lowest. Regarding to different zones, the highest mPBS was in coronal zone, and the lowest one was in the apical zone. The aging procedure also led to a statistically-significant decrease in mPBS. Most frequent failure modes were cohesive failure in dentin and mixed failure. Irrigation with 7%MA (Gr4) showed better performance than 17% EDTA (Gr3) in smear layer removal, especially at the apical zone of the tooth. This is critical for the success of root canal treatment and increased the mPBS to a higher extent in all zones of the tooth.

## Introduction

The restoration of teeth with coronal destruction used to be performed using cast metal posts and cores [1]. However, cast metal posts have some disadvantages, such as their inability to meet aesthetic expectations. Their rigid structure that is not compatible with the modulus of elasticity of the dentin that can cause root fracture [2, 3]. Fiber posts have recently become an appealing choice thanks to their aesthetically satisfactory properties and compatibility with the elasticity of the dentin. Nevertheless, the fact that fiber posts are prefabricated has caused problems in their adaptability with the morphology of the root canal [2]. In this way, CAD/CAM posts that combine the intra-canal adaptability of cast metal posts and the elastic modulus of fiber posts to dentin have come to the fore and have been introduced to the market [4, 5].

Adaptability between post and root canal is an important factor that increases the success of mechanical adhesion, and it is vital for the success of the treatment. Adhesion has two aspects as between dentin and resin cement and between resin cement and post [6]. Prior to post placement, dentin is subjected to various root canal irrigation protocols to strengthen adhesion [7]. While procedures such as sandblasting or exposure to hydrofluoric acid (HFA) are performed on posts [8, 9], procedures that are performed on the dentin include the application of various chelation solutions such as EDTA, citric acid (CA), maleic acid (MA), and chitosan (Ch). Chelation solutions are used to increase adhesion. They are used in conjunction with NaOCl, they can remove the smear layer on dentin and expose dentinal tubules. This way, the strength of the mechanical bond that will be established by sealer will be increase. Most commonly used chelation agent is EDTA and organic-containing agents such as CA, MA, Ch are also used [9, 10].

After cementation, restorations are exposed to various factors inside the mouth such as thermal effects, mechanical forces, and moisture. To imitate these conditions, in vitro studies utilize thermal or mechanical aging procedures. These procedures affect the bond strength between post and dentin [11, 12].

The shape formed by the canal and post preparation process has a conical structure that narrows towards the apical direction. For this reason, irrigation solution replacement in the apical region and removal of the smear layer from the dentin differ from the coronal to the apical direction. As a result, bond strength between dentin and gutta-percha or post varies in different zones of the root [13–15].

Various test methods are available for bond strength measurement [16]. One of the most frequently used among these is micro push out bond strength (mPBS) test [17]. It is thought that the mPBS test method measures the actual bonding efficiency more accurately than the traditional shear bond strength test. This is due to a parallel failure of the dentin-cement-post interface similar to the clinical situation when using mPBS [18]. In addition, due to the large number of early failures during sample preparation and the large amount of data associated with microtensile testing, the mPBS test has been considered to be more reliable than the microtensile test for dentin adhesion of posts [17, 19, 20].

The null hypothesis of our study was that different chelation agents and aging procedures do not affect the bond strength in different zones of the tooth (Coronal (C), middle (M) and Apical (A)).

### Ethics committee approval

The protocol of this study was reviewed in the meeting of the Hamidiye Scientific Studies Ethics Committee of the University of Health Sciences dated January 28, 2022 and found ethically appropriate (Document date - number: February 1, 2022–7253).

## Material and method specimen preparation

The material of the study consisted of 75 single-rooted and single-canalled mandibular premolar teeth (50 teeth (*n* = 10) to be used in the mPBS test and 25 (*n* = 5) to be used to examine the smear layer) extracted for orthodontic indications. The patients are adult orthodontic patients aged between 18 and 40 (average age 27), and their teeth were extracted after obtaining informed consent from the patients. A total of 300 samples, 6 slices from each root, were prepared for the mPBS test. After the patients filled out the informed consent, care was taken to ensure that the extracted teeth did not include root canal calcification, root caries, open apices, apical or cervical lesions, or internal or external root resorption, and teeth that did not meet these criteria were excluded. To confirm that each tooth had a closed apex and a single straight canal, two radiographic images were taken from each of the mesiodistal and buccolingual directions for each tooth. Dental calculus, periodontal ligament residues, and soft tissue residues on the teeth were scaled from the root surface with the help of a sharp periodontal curette (Hu-Friedy, Chicago, IL, USA). The teeth were stored in 0.5% Chloramine T solution at + 4 °C to be used within one month after extraction.

The crowns of the teeth were removed under distilled water cooling from at least 1 mm apical of the cement-enamel interface using a high-speed diamond disc (Diatech, Coltene AG, Switzerland), and the roots were measured using precision caliper and standardized to 16 mm in length. The roots were then cut from the 16 mm border using a high-speed diamond disc. Before preparation, to achieve a working length shorter than the actual root length by 1 mm (total working length: 15 mm), a [International Standardization Organization (ISO)] no. 10 K file (Dentsply/Maillefer, Ballaigues, Switzerland) was inserted into the canal with radiographic guidance. The canals were widened to ISO size no. 20 using K files for pre-enlargement. Next, using the Protaper Universal (PTU, Dentsply Sirona, Ballaigues, Switzerland) system files for root canal enlargement, starting from SX, the apical diameter was enlarged gradually up to no. 40 (F4). At each step of file change, the canals were irrigated with 2 ml 2.5% sodium hypochlorite (NaOCl) with a 30-gauge needle with a single side vented for 1 min. The canals were then dried with sterile paper points (Diadent Group International, Cheongju, Korea), and they were filled with gutta-percha and AH-plus root canal sealer (Dentsply DeTrey, Konstanz, Germany) using the lateral condensation technique. The coronal zones of the teeth were temporarily closed with resin composite (OmniChroma, Tokuyama Dental, Tokyo, Japan), and the teeth were stored in airtight containers at 37°C and 100% humidity for 7 days to simulate clinical conditions.

In the next step, the coronal fillings were removed, and the roots were prepared for the placement of posts. The post cavities were enlarged up to the 3rd drill size by removing the gutta-percha using drills in the post shaping system (3 M ESPE, Seefeld, Germany). Five millimeters of root canal filling was left in the apical direction to avoid disrupting the seal at the apical, and the post cavity that was created was standardized to 10 mm. The roots were then randomly divided into 5 groups (*n* = 10). In the groups, different combinations of various irrigants (saline solution [SS]; [NaOCl]; ethylenediaminetetraacetic acid [EDTA]; maleic acid [MA]; chitosan [Ch]) were used (Table 1). Each irrigation solution was applied at a volume of 5 ml for 1 min, and the canals were then dried using sterile paper points.

**Table 1:** Root canal irrigation protocols in the groups

Root Canal Irrigation Protocols	
Gr1	0.9% SS
Gr2	2.5% NaOCl + 0.9% SS
Gr3	17% EDTA + 2.5% NaOCl + 0.9% SS
Gr4	10% MA + 2.5% NaOCl + 0.9% SS
Gr5	0.2% Ch + 2.5% NaOCl + 0.9% SS

The post pattern was created using resin pattern and fiber post using modeling resin (Palavit G., Heraeus-Kulzer, Germany) in the post cavity, the materials were scanned using a CAD/CAM system (Ceramill, Amann Girrbach, Koblach, Austria) and modeled (Ceramill Mind Software). Then, from Vita Enamic (Vita Zahnfabrik, Bad Sackingen, Germany) (Hybrid ceramic) blocks, milling was performed with a volumetric reduction of 2.5% for uniform cementation using milling machine (Ceramill Motion 2, Amann Girrbach, Koblach, Austria) The milled CAD/CAM posts were subjected to surface roughening for 1 min using 9.6% Hydrofluoric acid (HFA) (Pulpdent), they were cleaned and dried using 70% ethanol + distilled water + air (60 s each), and silane (ANS, Porcelain Bonding Resin, Bisco, Schaumburg, IL, USA) was applied.

The self-etch-adhesive resin cement (Panavia F2.0 (Kuraray Medical, Inc., Okayama, Japan)) was injected into the root canal using endodontic tips, after conditioning the dentin with ED Primer II (A and B liquid, Kuraray Medical, Inc., Okayama, Japan) using a bond application brush for 60 s. The excess primer was moved away from cavity using paper points and the posts were cemented into the canals. The cement was polymerized by light curing for a total of 120 s, 30 s from each direction. Following cementation, the excess part of the post that remained outside the root was cut out, and the coronal zone of the root was completely sealed with composite resin.

After keeping the teeth in distilled water at 37°C for 7 days, each group was divided into 2 subgroups. BA, (before aging): Teeth on the 7th day after post placement.

AA, (after aging): Teeth to which aging was applied after the 7th day.

In the aging procedure, 1,000,000 mechanical cycles (50 N force was applied from the occlusal direction at 5 Hz frequency) were applied, corresponding to 5 years of aging [21].

## Micro push-out bond strength test

Fifty teeth were used for the mPBS test. They were randomly divided into 2 groups, where 25 teeth were subjected to aging, the other 25 teeth were not. The zones of the teeth were named as coronal (C), middle (M), and apical (A).

The teeth in all groups were embedded in self-curing acrylic resin, and blocks were obtained. The specimens were then placed on a precision cutter (Isomet, Buehler Ltd., Lake Bluff, NY, USA), and the roots were divided into 1-mm-thick slices under distilled water cooling. Including two slices from the coronal, two slices from the middle-third, and two slices from the apical of each root, 6 slices in total were obtained per tooth. The diameters of the posts on both sides of the slices and the thicknesses of all slices were measured using a digital caliper with an accuracy of 0.01 mm (Mitutoyo, Santo Amaro, SP, Brazil) and recorded.

Each slice was subjected to the mPBS test. The test was performed by applying a compressive load with a cylindrical punch pin connected to the device (DL-2000) at a rate of 0.5 mm/min from the apical to the coronal until the post was separated from the dentin. Punch pins at different diameters were used in different root zones, and the implementation was made by placing the pins at the center of the post surface.

The maximum failure load was recorded in N and converted to MPa. The MPa value was calculated by dividing the force (N) by the bonding surface (AL).

AL: The lateral area of the cylindrical post slice that is cut and sliced. The formula that was used to calculate AL was as follows:

$$\mathrm{AL}=\pi\left(R+r\right)\sqrt{h^2+\left(R-r\right)^2}$$

R: Coronal post diameter.

r: Apical post diameter.

h: Slice thickness.

For each group designated based on its tooth zones and whether the teeth in it were subjected to the aging process, a mean MPa value was calculated (separately for the C, M and A zones and for the BA and AA categories). These values were included in the statistical analyses.

## Modes of failure

**Fig. 1:**
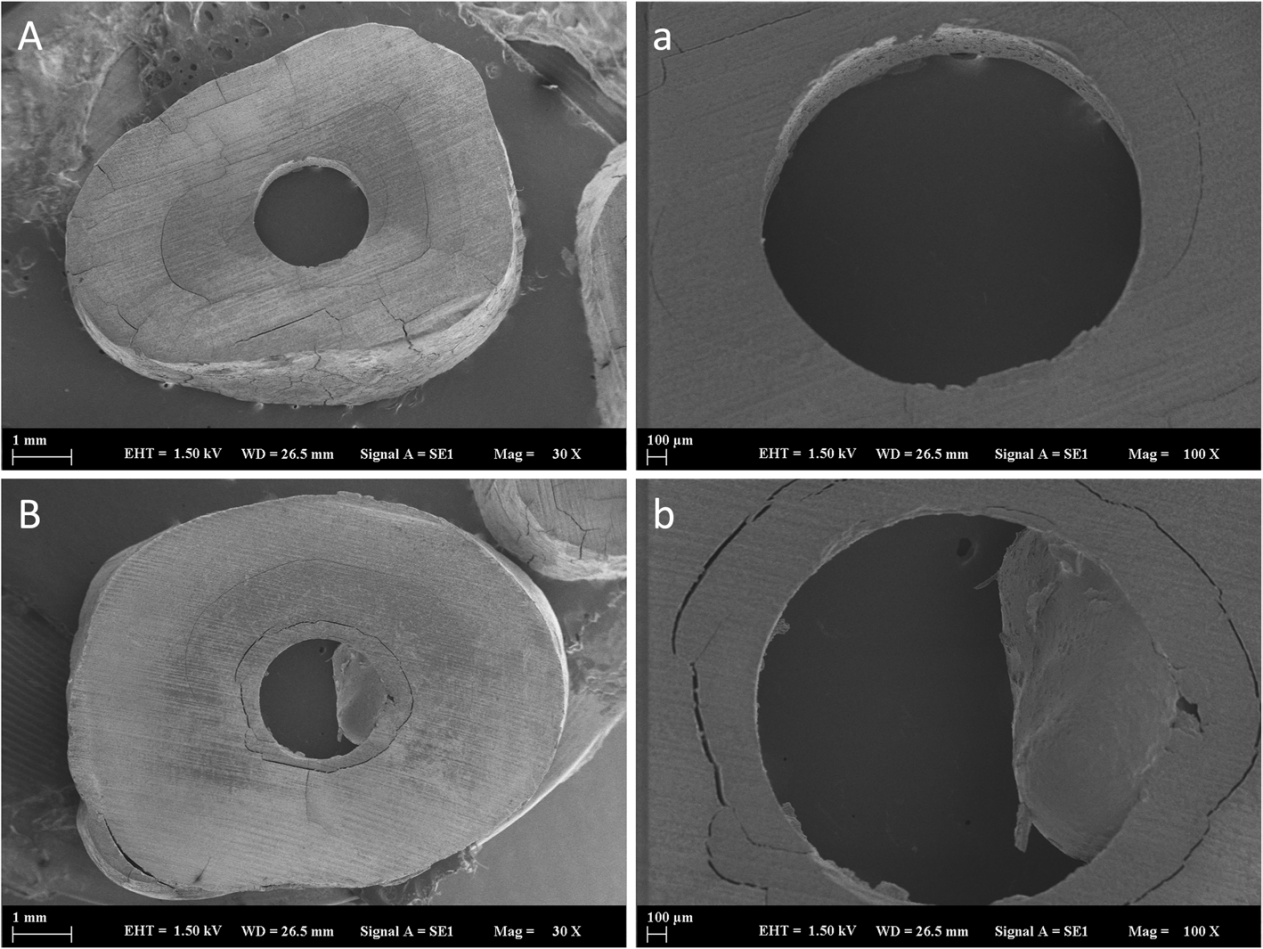
SEM images. Most frequent modes of failure (Type 3 and Type 5). Type 3 Adhesive in cement or dentin (A and a). Type 5 Mixed (B and b). 30x (A and B) and 100x (a and b). The lengths are given under the image as 1 mm (A and B) and 100µm (a and b)

Following the mPBS test, the slices were fixed on metallic clips, their surfaces were coated by spraying 200 Å gold powder (Polaron C502; Fisons Instruments, Uckfield, UK), and they were examined under a SEM. Modes of failure were examined at 30x and 100x magnification under a SEM (EVO LS-10, Zeiss, Cambridge, UK) (Fig. 1), and the examinations were interpreted based on a previous study as a reference [15].

## Smear layer examination

To examine remaining smear layer, a total of 25 (*n* = 5), were prepared for SEM assessments. Procedures such as root canal measurement, preparation, irrigation, root canal obturation, post-cavity preparation and irrigation protocol specific to the groups, which were performed for each group before the mPBS test, were also applied to the teeth to be used for the removal of the smear layer in the same way. After irrigation protocol (Table 1), each root was divided longitudinally along the labio-lingual direction and dehydrated using a graded ethanol series (in ascending order of concentration: 70%, 80%, 90%, and 100%). The specimens were then fixed on metallic clips, their surfaces were coated by spraying 200 Å gold powder, and they were examined under a SEM. On each level (i.e., zone: C, M, A), 10 representative images were taken for each root (up to 5000x magnification), and according to the criteria reported by Torabinejad et al. [19], the degree of smear layer removal was examined and scored by three blinded and calibrated observers.

### Statistical analysis

#### Statistical Method

The Minitab 17 program was used to analyze the data. The normality of data distributions was tested using the Shapiro-Wilk test. 3-way analysis of variance (ANOVA) was utilized to make comparisons based on the root canal irrigation protocols, tooth zones, and aging status, while Tukey’s HSD test was used for the multiple comparisons. The analysis results are presented as mean ± std. deviation for the quantitative data. The level of statistical significance was taken as *p* < 0.050 (Table 2).

**Table 2:** Bond strength comparison based on root canal irrigation protocols, zones, and aging status

	df	Sum of Squares	Mean Square	F	p
Root canal irrigation protocol	4	1481.510	370.377	2286.730	< 0.001
Zone	2	352.650	176.325	1088.640	< 0.001
Aging	1	121.290	121.285	748.820	< 0.001
Root canal irrigation protocol*Zone	8	13.320	1.666	10.280	< 0.001
Root canal irrigation protocol*Aging	4	2.040	0.509	3.150	0.015
Zone*Aging	2	0.020	0.009	0.050	0.948
Root canal irrigation protocol*Zone*Aging	8	1.150	0.144	0.890	0.526

*df*  Degrees of freedom, *F* ANOVA test statistic, R^2^ = 97.83%, Adjusted R^2^ = 97.60%

**Table 3:** Descriptive statistics and multiple comparison results on bond strength values based on root canal irrigation protocols, zones, and aging status

Root canal irrigation protocols	Aging	Zone			Total
		C	M	A	
Gr1 (0.9% SS)	Before (BA)	6.27 ± 0.42	5.83 ± 0.50	4.64 ± 0.53	5.58 ± 0.84^G^
	After (AA)	4.91 ± 0.15	4.30 ± 0.31	2.90 ± 0.42	4.04 ± 0.91^ H^
	Total	5.59 ± 0.76^ H^	5.07 ± 0.88^I^	3.77 ± 1.01^ J^	4.81 ± 1.17^a^
Gr2 (2.5% NaOCl + 0.9% SS)	Before (BA)	8.44 ± 0.34	7.15 ± 0.22	5.31 ± 0.49	6.97 ± 1.35^ F^
	After (AA)	7.40 ± 0.54	5.90 ± 0.15	4.40 ± 0.43	5.90 ± 1.31^G^
	Total	7.92 ± 0.69^ F^	6.53 ± 0.67^G^	4.86 ± 0.64^I^	6.43 ± 1.42^b^
Gr3 (17% EDTA + 2.5% NaOCl + 0.9% SS)	Before (BA)	10.76 ± 0.50	9.41 ± 0.41	8.12 ± 0.22	9.43 ± 1.16^D^
	After (AA)	9.60 ± 0.51	8.20 ± 0.29	6.90 ± 0.38	8.23 ± 1.19^E^
	Total	10.18 ± 0.77^CD^	8.81 ± 0.71^E^	7.51 ± 0.70^ F^	8.83 ± 1.31^c^
Gr4 (10% MA + 2.5% NaOCl + 0.9% SS)	Before (BA)	13.48 ± 0.48	11.60 ± 0.44	10.29 ± 0.39	11.79 ± 1.40^ A^
	After (AA)	12.10 ± 0.37	10.40 ± 0.53	9.30 ± 0.44	10.60 ± 1.25^B^
	Total	12.79 ± 0.82^ A^	11.00 ± 0.78^B^	9.80 ± 0.65^D^	11.20 ± 1.44^d^
Gr5 (0.2% Ch + 2.5% NaOCl + 0.9% SS)	Before (BA)	11.14 ± 0.25	9.67 ± 0.39	8.48 ± 0.39	9.76 ± 1.16^ C^
	After (AA)	9.80 ± 0.42	8.40 ± 0.36	7.00 ± 0.37	8.40 ± 1.22^E^
	Total	10.47 ± 0.76^ C^	9.04 ± 0.75^E^	7.74 ± 0.84^ F^	9.08 ± 1.36^e^
Total	Before (BA)	10.02 ± 2.52	8.73 ± 2.08	7.37 ± 2.16	8.71 ± 2.49
	After (AA)	8.76 ± 2.49	7.44 ± 2.17	6.10 ± 2.28	7.43 ± 2.55
	Total	9.39 ± 2.57^a^	8.09 ± 2.21^b^	6.73 ± 2.30^c^	8.07 ± 2.60

^a-e^No significant difference between main effects with the same letters, ^A-J^No significant difference between interaction effects with the same letters.

## Results

The results of the bond strength comparison based on root canal irrigation protocols, zones, and aging status are shown in Table 2. Table 3 represents the descriptive statistics and multiple comparison regarding bond strength based on the root canal irrigation protocols, zones, and aging status.

The main effect of the root canal irrigation protocol variable on bond strength was found statistically significant (*p* < 0.001). The mean bond strength values were 4.81 ± 1.17 MPa in Gr1, 6.43 ± 1.42 MPa in Gr2, 8.83 ± 1.31 MPa in Gr3, 11.20 ± 1.44 MPa in Gr4, and 9.08 ± 1.36 MPa in Gr5. While the highest mean bond strength value was obtained in Gr4, the lowest one was obtained in Gr1.

The main effect of the zone variable on bond strength was found statistically significant (*p* < 0.001). The mean bond strength was 9.39 ± 2.57 MPa in zone C, 8.09 ± 2.21 MPa in zone M, and 6.73 ± 2.30 MPa in zone A. While the highest mean bond strength was obtained in zone C, the lowest one was obtained in zone A.

The main effect of the aging status variable on bond strength was found statistically significant (*p* < 0.001). The mean bond strength was 8.71 ± 2.49 MPa BA and 7.43 ± 2.55 MPa AA. The mean bond strength in the BA specimens was significantly higher than that in the AA specimens.

The interaction effect of the root canal irrigation protocol and zone variables on bond strength was found statistically significant (*p* < 0.001). While the highest mean bond strength was obtained in zone C in Gr4 as 12.79 ± 0.82 MPa, the lowest one was obtained in zone A in Gr1 as 3.77 ± 1.01 MPa.

The interaction effect of the root canal irrigation protocol and aging status variables on bond strength was found to be statistically significant (*p* = 0.015). While the highest mean bond strength value was obtained in the BA specimens in Gr4 as 11.79 ± 1.40 MPa, the lowest one was obtained in the AA specimens in Gr1 as 4.04 ± 0.91 MPa.

The interaction of zone and aging status variables and the interaction of the root canal irrigation protocol, zone, and aging status variables did not have a statistically significant effect on bond strength (*p* > 0.050). The multiple comparison results on the main effects and interaction effects are shown with letters in Table 3.

## Modes of failure

**Fig. 2:**
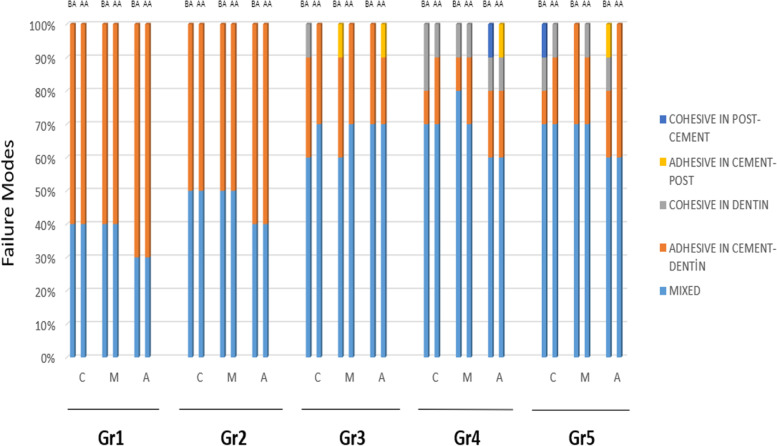
Distribution of modes of failure based on groups. Type 1: Cohesive in dentin. Type 2: Cohesive in post or cement. Type 3: Adhesive in cement or dentin. Type 4: Adhesive in cement or post. Type 5: Mixed

The failure modes were categorized as follows: 1, Cohesive in dentin; 2, Cohesive in post or cement; 3, Adhesive in cement or dentin; 4, Adhesive in cement or post; 5, Mixed. Most frequent modes of failure were Type 3 and Type 5 (Fig. 2).

The graph in Fig. 2 shows the rates of modes of delivery observed in the groups (G1-5), zones (C, M, A), BA, and AA.

## Smear layer examination

**Fig. 3:**
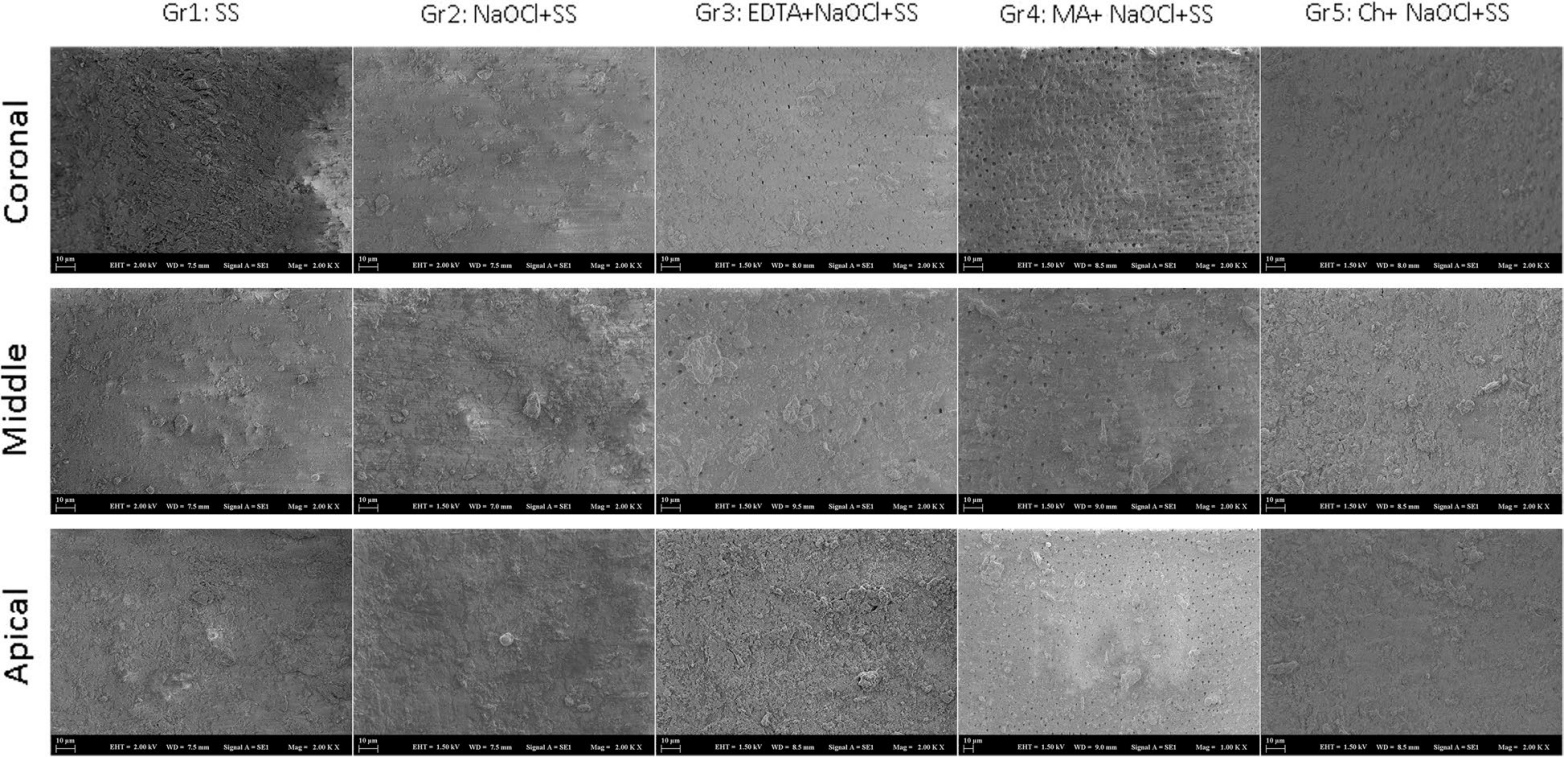
SEM images in groups and zones

The scoring system for smear layer removal was as follows: 1, no smear layer (no smear layer on the surface of the root canal, and all tubules are clean and open); 2, moderate smear layer (no smear layer on the surface of the root canal, but tubules contain debris); 3, heavy smear layer (smear layer covers the root canal surface and the tubules) (Table 4). The most successful protocol, which removes the smear layer in all root zones and especially in the apical zone, was found in the irrigation protocol of Gr4. (Fig. 3).

**Table 4:** Smear layer scores of the Scanning Electron Microscopy (SEM) images

	Gr1	Gr2	Gr3	Gr4	Gr5
C	**3**	**3**	**2**	**1**	**2**
M	**3**	**3**	**2**	**2**	**2**
A	**3**	**3**	**3**	**2**	**3**

## Discussion

In our study, instead of metallic posts, posts milled out of Vita Enamic blocks with a similar modulus of elasticity to that of the dentin were used. The modulus of elasticity values of metal posts that have been frequently used from the past to the present are much higher than that of the dentin [20]. Therefore, due to this incompatibility between the modulus of elasticity of metal post and dentin, various complications such as secondary caries, discoloration, decementation, cracks and fractures are encountered in dentin [22]. On the other hand, because metal posts are produced using the method of root canal duplication and metal casting, their adaptability with the shape of root canal is higher. The difference of elastic modulus between metal posts and dentin can cause root fracture, therefore prefabricated fiber posts with closer elastic modulus to dentin are used. However, one of the disadvantages of fiber posts is poor adaptability to root canal’s anatomy. As a result of this inadaptability, gaps can be present between post and dentin, and micro-leakages can occur  [2, 23]. Posts made from Vita Enamic, a polymer infiltrated ceramic network, are milled out of blocks using post pattern created directly from the root canal. Therefore, post fabricated with this material and method can achieve good adaptability and high compatibility with the elastic modulus of dentin [2, 3, 20, 21].

Adhesion is one of the most significant factors that affect success in the long-term prognosis of a tooth that is treated by post and core. Adhesion has two aspects, one between dentin and cement, and the other between cement and post [11]. Post and dentin are subjected to various root canal irrigation protocols to strengthen adhesion. While procedures such as sandblasting or exposure to HFA are performed for the post, dentin surfaces can be treated with various chelation agents [9, 10]. In our study, HFA was applied to the surface of the post, while different irrigation and chelation agents were applied to the surface of the dentin in each group. From the past to the present, various irrigation and chelation agents such as NaOCl, EDTA, and citric acid have been applied to the dentin’s surface.

Some studies have shown that the bond strength between bondings and dentin is reduced when NaOCl is used as the final irrigation agent [24, 25]. Therefore, saline solution was used in our study in order not to adversely affect the bonding and to remove NaOCl from the surface. After removing the NaOCl from the surface, the dentin was dried with sterile paper points and bonding agent (ED Primer II (A and B liquid, Kuraray)) was applied. The self-etch resin cement was used in this study. In this study, more biocompatible and organic agents like MA and Ch were also used in addition to conventional chelation agent. This study also examined the effects of these solutions on the bond strength between the post and the dentin in different zones of the root canal and whether this bond strength changed after aging procedure was applied. As a result of this study, the null hypothesis was rejected.

Different root canal irrigation solutions, different zones of the tooth, and the aging process had a statistically significant effect on mPBS (Table 3). SEM images taken from the root surfaces and smear layers on the surfaces were examined and scored (Table 4). According to this scoring process, the root surfaces in Gr1 and Gr2 were completely covered in smear layers, the surfaces in Gr3 and Gr5 contained moderate smear layers, and the surfaces in Gr4 were relatively cleaner with less smear layer. Some studies reported that the effectiveness of MA in removing the smear layer from root canal surfaces is very high, and it provides even better results than EDTA [13, 14]. In our study, Gr4 involving the use of MA provided better results than the other groups in terms of removing the smear layer and achieving bond strength. Moreover, it was observed that Gr3 and Gr5 showed a similar removal of the smear layer, and they had a similar effect on the mPBS. Kesim et al. investigated sealer penetration into the dentinal tubules using the confocal laser scanning method, they did not find a significant difference among EDTA, Ch, and CA [26].

The main reason for this study to apply aging was to imitate the dynamic intraoral environment and determine the solution for the best long-term prognosis after post treatment and preserve bond strength for a longer period. In the comparisons of all root canal irrigation protocols and root zones based on the aging process, a statistically significant difference was found between BA and AA. The highest mPBS values were obtained when BA was used, while the lowest ones were obtained when AA was applied. These results were compatible with some previously reported results [27, 28].

The interaction of root canal irrigation protocol and aging status on bond strength was found statistically significant. While the highest mean bond strength was obtained in Gr4 applying MA for the BA specimens, the lowest mean bond strength was in Gr1 by applying SS for the AA specimens. The more effective removal of the smear layer by MA in comparison to the other solutions may have played a role in the increase in mPBS [13]. MA provided a similar smear layer removal efficacy to EDTA and Ch in the C and M zones, but it was more effective compared to the others in zone A [19, 29, 30]. It is crucial to ensure that the root canal preparation diameter at the apical area was enlarged as it would increase the effectiveness of irrigation in this region [31–33]. In this study, root canal instrumentation was done using F4 rotary files with apical diameters of ISO no. 40. In comparison to MA, EDTA could not remove the smear layer as effectively. The reason for this may be the fact that the surface tension of 17% EDTA (0.0783 N/m) is higher than the surface tension of MA (0.06345 N/m) [11]. Additionally, the more effective smear layer removal characteristic of MA compared to EDTA in the apical zone of the root canal may be explained by its strong acidic properties that induce a rapid demineralizing effect in a short time. Similarly, some researchers have reported the present of sclerotic detin in the apical zone of the root canal [20, 34]. Furthermore, EDTA may lose its effectiveness due to the decrease in its pH over time due to calcium and hydrogen exchange during its reaction with the dentinal surface [35, 36]. In this study, the group that was irrigated with Ch (Gr5) showed similar results to those in the EDTA group (Gr3). Our results were in agreement with the results of other studies [37, 38].

Regarding the effect of root canal irrigation and bond strength, the highest bond strength value was obtained in Gr4, where MA was used for irrigation. The second-highest value was obtained in the Ch group, and the lowest value was obtained in the control group (Gr1), where saline was used for irrigation.

According to the results of this study, the effect of MA on bond strength was significantly higher than the effects of the other solutions. The bond strength in the control group (Gr1, canals irrigated with saline) was significantly lower, and the SEM analysis of Gr1 showed almost no effect in smear layer removal. This result was in parallel with the results reported by Carvalho et al. [39]. In this study, the main effect of the zones (C, M, A) on bond strength was statistically significant. While the highest mean bond strength value was obtained in zone C, the lowest one was obtained in zone A. In their study on AH Plus penetration, Camilleri [40] reported that there was penetration into the dentinal tubules in zones C and M, but penetration did not always occur in zone A. In addition to the larger number of dentinal tubules in zones C and M, the larger tubule diameters compared to zone A [41] may have led to better sealer penetration [42, 43]. This may explain why highest bond strength in zone C was achieved due to the higher degree of micro-tag and mechanical bond formation.

For mode of failure, it was determined that most specimens had mixed-type (Type 5) failures, and the second-most frequently encountered mode of failure was an adhesive between cement and dentin (Type 3) (Figs. 1 and 2). While Type 3 failures were frequently seen in Gr1 and Gr2, the rates of mixed-type failures increased in Gr3, Gr4, and Gr5. The absence of chelation agents in Gr1 and Gr2 resulted in the minimal removal of the smear layer in comparison to the other groups, or none at all (Table 1; Fig. 3). Thus, in these cases, the bond strength may have been lower than those in the other groups depending on the inadequate formation of micro-tag bonds with the dentinal tubules. This could explain the Type 3 failures (adhesive in cement or dentin) in these groups, where sealer (i.e., cement) was completely separated from dentin. In the other groups, with the usage of chelation agents, the smear layer was removed more effectively. The increased bond strength between dentin and sealer may have led to an increase in the rate of mixed-type (Type 5) failures (Fig. 1).

## Conclusion

Application of Maleic acid was found to be significantly superior to the other root canal irrigation protocols in all zones of the root canal for removing smear layer and increasing micro push-out bond strength. The use of normal saline solution and Sodium hypochlorite were found to be less successful when compared to other. Limitations of this study include the fact that this was an in vitro study, and the intraoral environment could not be replicated entirely. Therefore, it is recommended to support the results of this study with long-term clinical studies.

## Data Availability

The data analyzed in the present study are available in related file.
